# Proposal for a Five-Step Method to Elicit Expert Judgment

**DOI:** 10.3389/fpsyg.2017.02110

**Published:** 2017-12-05

**Authors:** Duco Veen, Diederick Stoel, Mariëlle Zondervan-Zwijnenburg, Rens van de Schoot

**Affiliations:** ^1^Department of Methods and Statistics, Utrecht University, Utrecht, Netherlands; ^2^ProfitWise International B.V., Amsterdam, Netherlands; ^3^Optentia Research Focus Area, North-West University, Potchefstroom, South Africa

**Keywords:** Bayesian statistics, elicitation, expert judgment, expert knowledge, Five-Step Method, prior

## Abstract

Elicitation is a commonly used tool to extract viable information from experts. The information that is held by the expert is extracted and a probabilistic representation of this knowledge is constructed. A promising avenue in psychological research is to incorporated experts’ prior knowledge in the statistical analysis. Systematic reviews on elicitation literature however suggest that it might be inappropriate to directly obtain distributional representations from experts. The literature qualifies experts’ performance on estimating elements of a distribution as unsatisfactory, thus reliably specifying the essential elements of the parameters of interest in one elicitation step seems implausible. Providing feedback within the elicitation process can enhance the quality of the elicitation and interactive software can be used to facilitate the feedback. Therefore, we propose to decompose the elicitation procedure into smaller steps with adjustable outcomes. We represent the tacit knowledge of experts as a location parameter and their uncertainty concerning this knowledge by a scale and shape parameter. Using a feedback procedure, experts can accept the representation of their beliefs or adjust their input. We propose a Five-Step Method which consists of (1) Eliciting the location parameter using the trial roulette method. (2) Provide feedback on the location parameter and ask for confirmation or adjustment. (3) Elicit the scale and shape parameter. (4) Provide feedback on the scale and shape parameter and ask for confirmation or adjustment. (5) Use the elicited and calibrated probability distribution in a statistical analysis and update it with data or to compute a prior-data conflict within a Bayesian framework. User feasibility and internal validity for the Five-Step Method are investigated using three elicitation studies.

## Introduction

“*The knowledge held by expert practitioners is too valuable to be ignored. But only when thorough methods are applied, can the application of expert knowledge be as valid as the use of empirical data. The responsibility for the effective and rigorous use of expert knowledge lies with the researchers.*” (Drescher et al., 2013, p. 1)

According to O’Hagan et al. (2006) elicitation is the process of extracting and creating a representation of an expert’s beliefs. It can be used for a variety of reasons, e.g., to add information to small sample data (Kadane, 1994; Zondervan-Zwijnenburg et al., 2017a; van de Schoot et al., in press), when there is no data for certain confounding parameters in a model (Fischer et al., 2013), when no data is available (Ho and Smith, 1997; Hald et al., 2016), as sensitivity analysis to check assumptions about missing data (Mason et al., 2017), or simply to enrich the available data (Wiśniowski et al., 2014). Expert knowledge is a valuable source of information, as becomes evident in the quote of Drescher. Hadorn et al. (2014) found that 57% of health economic decision models included at least one expert knowledge elicitation parameter, showing that in some fields it is even the norm to use expert elicitation. More examples of elicitation practices in many different fields can be found in overview studies by O’Hagan et al. (2006, chap. 10) and Bistline (2014) or the paper by Cooke and Goossens (2008) in which they describe the data base of over 67,000 experts’ subjective probability distributions.

There are many elicitation procedures available, overviews can be found in for instance O’Hagan et al. (2006), Johnson et al. (2010a), and Aspinall and Cooke (2013). A popular elicitation method is the trial roulette method (Gore, 1987), sometimes also called the chips and bins method or the histogram method, in which experts assign “chips” to “bins” of a histogram to ascribe probability. In the procedure, used by for instance Diamond et al. (2014) and Goldstein and Rothschild (2014), the parameter space for which experts can assign probability is divided into equal sections or “bins.” The experts receive 20 “chips,” which are to be distributed amongst these “bins.” For each “chip” that is allocated to one of the “bins,” 5% of the mass of a probability distribution is ascribed. Based on the input provided by the expert, a probability distribution is fitted. The trial roulette method has been validated by Johnson et al. (2010b) and Zondervan-Zwijnenburg et al. (2017b) in a face-to-face setting.

Software that can be used in the elicitation with the trial roulette method is available in the MATCH Uncertainty Elicitation Tool (Morris et al., 2014). MATCH is an online framework for elicitation procedures. It uses the R-package (R Core Team, 2014) SHELF (Oakley, 2016) to fit appropriate parametric distributions based on input that is provided by experts.

One of the reasons the trial roulette method is popular is that the procedure provides immediate visual feedback to experts. Feedback is important in elicitation procedures to reduce bias and improve the quality of the elicitation (O’Hagan et al., 2006; Johnson et al., 2010a). The “chips” that are allocated in the trial roulette method by the expert visually approximate a probability distribution. However, the feedback provided to the expert is not on the statistical distribution that is actually used by the researcher in the final analyses. It is important to receive conformation of the expert that the interpretation by the researcher matches their beliefs, or as O’Hagan et al. (2006, p. 174) state, “*feedback to the expert is the most natural way of evaluating the distribution – the expert is in the best position to judge whether something corresponds to her opinion.”* Providing instant feedback on the representation of the experts’ beliefs, based on the input they provide, and how their beliefs are translated into a statistical distribution can easily be done by using software.

Feedback is believed to improve the quality of the elicitation procedure by making experts; reflect and maintain self-consistency (Fisher et al., 2012), by highlighting inconsistencies in judgment and making errors apparent (O’Hagan et al., 2006; Morris et al., 2014) and by allowing for self-correction by experts (Johnson et al., 2010a). Despite assumed quality improvement by feedback, systematic reviews on elicitation literature by O’Hagan et al. (2006) and Johnson et al. (2010a) conclude that measurement properties of elicitation methods have not been adequately evaluated. Moreover, there is no direct research into how accurate experts can assess properties like the mean, mode, or variance for the distribution of an uncertain parameter. Research by Johnson et al. (2010b) and Zondervan-Zwijnenburg et al. (2017b) provide promising results concerning the trial roulette method. Yet, directly obtaining distributional representations may be inappropriate given experts’ unsatisfactory performance on specifying elements of this distribution. O’Hagan et al. (2006) refer to research by Hofstatter (1939), Lathrop (1967), and Beach and Scopp (1968) to show that experts are not good at interpreting and assigning numerical values to variances and relative variability. It might then be unreasonable to assume that experts are able to reliably specifying a probability distribution in one step.

Therefore, to assist experts in the process of creating a representation of their beliefs in a statistical distribution we propose to decompose the elicitation task in smaller steps to encourage and assist in structured reasoning. Decomposing a problem into more tractable and familiar components is suggested by for instance Fischhoff (1982) to decrease the mismatch between the judge and the task. By decomposing the elicitation task we aim to reduce bias and incorporate more feedback to ensure that experts’ opinions are properly calibrated and represented by the probability distributions that results from the elicitation. In the current paper, the statistical distribution of interest is the skewed normal (SN) distribution^1^ because uncertainty might typically not best be captured by a symmetric distribution. This (un)certainty is the key feature of Bayesian statistics, uncertainty reveals the extent of our knowledge and ignorance (de Finetti, 1974).

We propose the Five-Step Method which consists out of the following steps:

(1) Elicit the location parameter of the SN using the trial roulette method.
(2) Use software to provide instant feedback on the interpretation of the expert’s beliefs by the researcher so the expert can accept this representation or adjust their input.
(3) Elicit the (un)certainty of the expert by determining the scale and shape parameters of the SN using expert’s statements on the lower and upper bounds for a plausible range of the parameter values.
(4) Use software to provide instant feedback on the interpretation of the expert’s (un)certainty about the location parameter by the researcher so expert can accept this representation or adjust their input.
(5) Use the elicited and calibrated probability distribution in a Bayesian analysis to update it with data or to compute a prior-data conflict.

The remainder of the paper is ordered as follows. We first provide details on the Five-Step Method. Thereafter we present a user feasibility study in which we elicited beliefs regarding a trivial sports related question from respondents to investigate visual and procedural preferences of users for the digitized version of the trial roulette method. A second study was carried out by asking experts working at a staffing company about certain key performance indicators which we used to validate the internal validity of steps 1 and 2 of the elicitation procedure. A final study was done with regional directors working at a large financial institution. They provided actual forecasts concerning average turnover per professional in the first quarter of the year 2016 with the Five-Step Method. The participating companies already make predictions concerning the parameters we elicit, yet they do this in the form of point estimates. The experts are thus already used to thinking about these data and predicting these data which makes them highly suitable to include as experts in an elicitation exercise. Yet, it is an extension for them to actively specify and separate knowledge and uncertainty. Because the companies also provided us with data on the predicted parameters we were able to compare the forecasts of the experts with data and thereby get an indication of the internal validity of the elicitation procedure. The proposition to split the elicitation process results in a procedure differing from the existing elicitation procedures as, for example, proposed by Oakley (2010), or that can be carried out through the use of existing software like MATCH. Therefore, we programmed our own software. All related materials for this study, including code and data, can be found on the Open Science Framework (OSF) webpage at https://osf.io/wvujz.

## Five-Step Method

In this section we describe the technical details of the Five-Step Method which has been programmed in R (R Core Team, 2014) using the shiny package (Chang et al., 2015).

### Step 1

**FIGURE 1:**
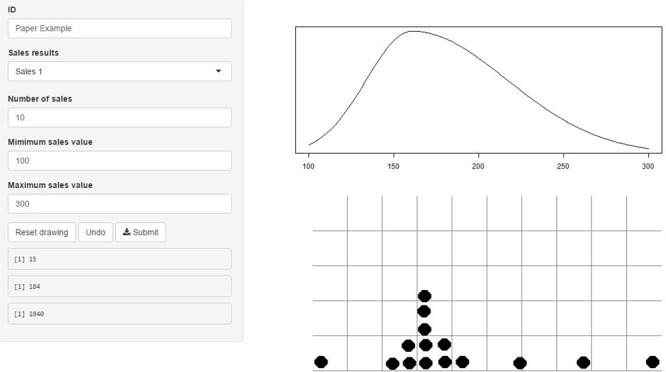
Shiny application for steps 1 and 2. On the left the input fields can be found for the reasonable lower and upper bound as minimum and maximum values. The input grid in which “chips” can be placed is found on the lower right with the leftmost dot being the minimum value and the right most dot being the maximum value. Further “chips” are placed by clicking the mouse drawing a maximum of 11 pixels left and right. On the top right feedback is provided, presenting the fitted distribution based on the input.

The first step of the Five-Step Method consists of a digitized version of the trial roulette, which can be seen in **Figure 1**. Instead of vertical “bins” a grid is used and the digital “chips” can be placed on the grid. Experts provide estimates for the expected minimum and maximum value of the parameter of interest, represented by the left and rightmost digital “chips” in the grid, based on which the range of the grid is determined. Thereafter they place additional “chips” in the grid. In specific, the input grid, denoted by **G**, is a matrix size 600 (columns) × 300 (rows) and cells are activated by the placement of a digital “chips” in the grid. The cells where a sticker is placed obtain a value of one, all other cells are set to non-available. A second matrix, denoted by **R** of the same dimensions is created in which all rows are equal and the columns are a sequence of numbers with equal intervals running from the reasonable lower to upper bound provided as input. We then create output matrix **O** which contains values from **R** activated by the placement of dots in **G** and after the deletion of all non-available values in **O**, the remaining values are stored in a vector.

### Step 2

The vector of values that is elicited in step 1 are used to fit a SN distribution. The SN distribution is defined in this paper as a normal distribution with the additional shape parameter γ. The shape parameter is based upon a general method for the transformation of symmetric distributions into skewed distributions as described in Fernandez and Steel (1998). The transformation of the symmetric distribution into a skewed distribution is done by allocating mass of the distribution to either side of the mode (M) by controlling the error term (𝜖) via the following function, taken from Fernandez and Steel Eq. 1:

(1) $$\begin{array}{c} p(𝜖|\gamma )\;=\;\frac{2}{\gamma \;+\;\frac{1}{\gamma }}\;\left\{f\left(\frac{𝜖}{\gamma }\right)I_{\left(M,\infty \right)}(𝜖)\;+\;f(\gamma 𝜖)I_{\left(-\infty ,M\right)}(𝜖)\right\}. \end{array}$$

**FIGURE 2:**
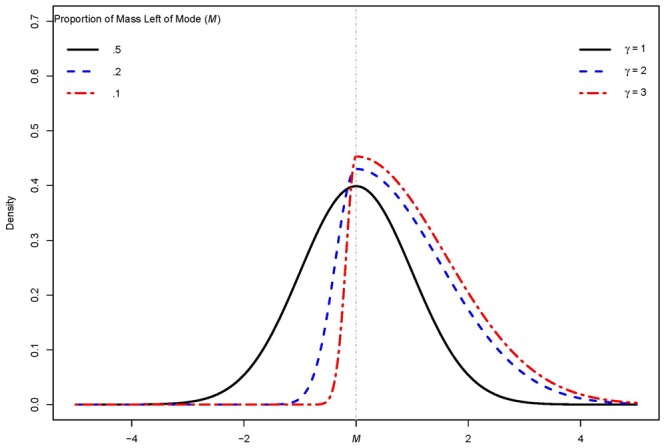
Example of the influence of shape parameter γ on the allocation of mass for a normal distribution with a variance of 1.

The effect of the shape parameter on the allocation of mass can be seen in **Figure 2**. Note that the distributions would be exactly mirrored with respect to the mode if the γ values would be $\frac{1}{\gamma }$.

To fit the SN distribution we make use of the snormFit function from the fGarch package (Wuertz et al., 2013). This function uses an optimization algorithm to determine the optimal skewness parameter based on log-likelihood values. The mean and standard deviation are determined based on the vector of elicited values. The mean and standard deviation remain constant and thus there is only one parameter to optimize over, the shape parameter γ .

The SN distribution that is fitted based upon the expert’s input is provided as visual feedback to the expert, see **Figure 1**. The visual feedback indicates how we interpret the information that is provided by the expert. The expert can accept the representation of their beliefs or adjust input until the representation matches their beliefs. Once the expert approves the representation of their beliefs, the mean value is extracted from the distribution which is to be used in step 3.

### Step 3

**FIGURE 3:**
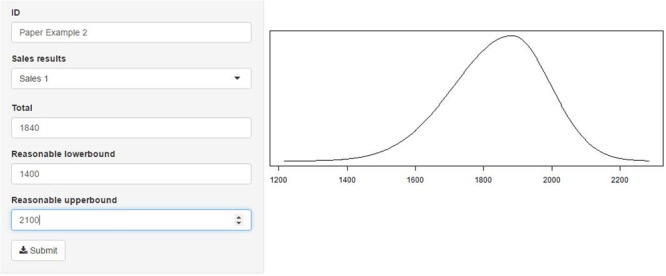
Shiny application for steps 3 and 4. On the left the input fields require entering the estimate for μ_0_ and the reasonable lower and upper bound for the estimate. On the right the distribution that is fitted based on the input can be found.

Step 3 of the Five-Step Method is used to derive the distributional representation of the expert’s prior beliefs concerning the parameter of interest and can be seen in **Figure 3**. We restricted the priors that represent the experts’ beliefs to be SN distributions so π_d_(𝜃) ∼ SN(μ_0_,$\sigma_{0}^{2}$,γ_0_), where subscript *d* denotes expert *d* = 1,…,*D*, μ_0_ denotes the prior mean, $\sigma_{0}^{2}$ denotes the prior variance, and γ_0_ denotes the prior skewness. The value for μ_0_ is assumed to be known, either obtained through steps 1 and 2 or stated directly. In step 3 the expert is required to provide values for the reasonable lower and upper bounds they perceive as likely for their estimate of μ_0_ The value for μ_0_ is repeated 100 times, the values for the reasonable lower and upper bounds for the estimate are both repeated 10 times.

### Step 4

Based on the input provided in step 3 we will obtain estimates for the scale parameter $\sigma_{0}^{2}$ and the shape parameter γ_0_. The 120 values, μ_0_ repeated 100 times and the values for the reasonable lower and upper bounds both repeated 10 times, are provided to the snormFit function by means of which a SN distribution is fitted. The estimates for $\sigma_{0}^{2}$ and γ_0_ are obtained and μ_0_ is constrained to the input value. Visual feedback is provided to the expert of the resulting SN distribution, which can be seen in **Figure 3**. The expert can accept the representation of their beliefs or adjust input until the representation matches their beliefs.

### Step 5

Use the elicited distribution that represents the expert’s beliefs.

## Elicitation Studies

In this section we describe the three studies we conducted. During the user feasibility study R version 3.1.2 was used and R version 3.2.3 was used during the elicitations done with the staffing company and the large financial institution. We conducted the elicitations in a semi-structured face-to-face setting so that the researcher could provide interpretations accompanying the visual feedback. An advantage of a face-to-face setting is that it allows clarification of procedural and elicitation related questions thereby improving the validity of the responses (O’Hagan et al., 2006).

Cooke and Goossens (1999) describe that a panel of four experts can be sufficient for an elicitation, but they recommend a panel of about eight experts as a rule of thumb. In the user feasibility study nine respondents participated. In the staffing company only four experts were available in the entire company, therefore the sample was limited to a size of four. Regarding the study at the large financial institution four experts participated in the end.

### User Feasibility Study

#### Design

With the user feasibility study we evaluated the usability of the first two steps of the Five-Step Method. Procedural and visual preferences were investigated. Four variations of the shiny application were tested. The respondents (*D* = 9), obtained through convenience sampling from a population of university trained adults, were randomly allocated to two out of the four possible variations of the software.

In the first procedural option, we used the procedure of the trial roulette where 20 digital “chips,” starting with the expected minimal and maximum value, each representing five percent of a distribution, were to be placed in a grid following the procedure described by Zondervan-Zwijnenburg et al. (2017b). After placing 20 “chips” the respondents could submit their input and they were provided with visual feedback on the distribution that was fitted based on these 20 “chips.” They could accept the representation or adjust their input. The second procedural option required the placement of a minimum of seven “chips,” starting with the expected minimal and maximum value. In this procedural variation the distribution that was fitted based on the input was constantly shown. The distribution changed with each placed “chip” and thus instant feedback was provided on the representation of the input. Respondents could, after placing a minimum of seven “chips,” at each point accept the representation of their beliefs or add or adjust input. Next to these two options, we also varied the size of the digital grid in which the “chips” were placed: large and small.

The respondents evaluated the two variations they were appointed to with a questionnaire asking if the fitted distribution was a good reflection of their beliefs and what visual and procedural preferences were. Additional questions were based on the taxonomy of Bloom et al. (1956) to identify weak points of the software and procedures. These questions investigated; the comprehension of the instructions, the ability to apply the tool, the understanding of the representation of the “chips,” the relation between input and fitted distribution, and the relation between belief and fitted distribution. The full questionnaire can be found in the data archive which is available on the OSF webpage at https://osf.io/wvujz.

#### Results

All respondents indicated that their beliefs where accurately represented. Five of the seven respondents allocated to both procedural variants preferred the second variation. Four of the six respondents allocated to both visual variants preferred the large grid, one abstained from answering. Three out of the nine respondents indicated for at least one of the variations that they did not understand the meaning of the “chips.” In the first procedural option the “chips” each represented 5% of the data whilst in the second procedural option the meaning depended on the amount of chips that were placed. They allocated mass for the distribution that was fitted. The meaning of the chips was not completely understood by one person who used the first procedure and by two persons who used the second procedure. All three of them used a small grid variation. The three respondents all indicated that they knew what the distribution representing their opinion meant in the end and agreed that this accurately described their view. Based on the results we decided to continue working with the second procedural variation, requiring the minimal placement of seven “chips” without further restriction on the number of “chips,” and a large grid.

### Elicitation Staffing Company

#### Design

The goal of the second study was to test the internal validity of elicitations obtained with the first two steps of the Five-Step Method. We found a staffing company willing to participate with experts (*D* = 4) providing predictions about five sales results concerning the first quarter of 2016: contract hours, hourly cost buying and selling, turnover and hourly sales margin. A staffing company is a link between companies that want to hire staff and staff looking to work at companies. They buy work from individuals and thereafter place them to work at other companies. The amount of hours they place an individual at another company in the quarter are the contract hours. The hourly cost buying is what it will cost them per hour to buy the work from the individuals and the hourly cost selling is the price which they charge the companies where they stall the individuals. The turnover is equal to the contract hours multiplied by the hourly cost selling and the hourly sales margin is equal to the hourly cost selling minus the hourly cost buying.

The experts were asked to predict the distribution of the data. In some sectors staffing companies staff a lot of individuals at low margins and thus generate a large turnover. In different sectors they staff few individuals at high margins thereby obtaining the same profit at lower turnover rates. These are all relevant considerations and the experts should know which is the case for their company. The company provided us with actual budgets they made which were indications of carefully constructed predictions. By comparing the predictions of the experts to the budget we could gain an indication of the internal validity of predictions made with the first two steps of the Five-Step Method. If the elicitation results match the budget this indicates that the procedure is able to represent the underlying construct of carefully constructed predictions.

#### Results

**FIGURE 4:**
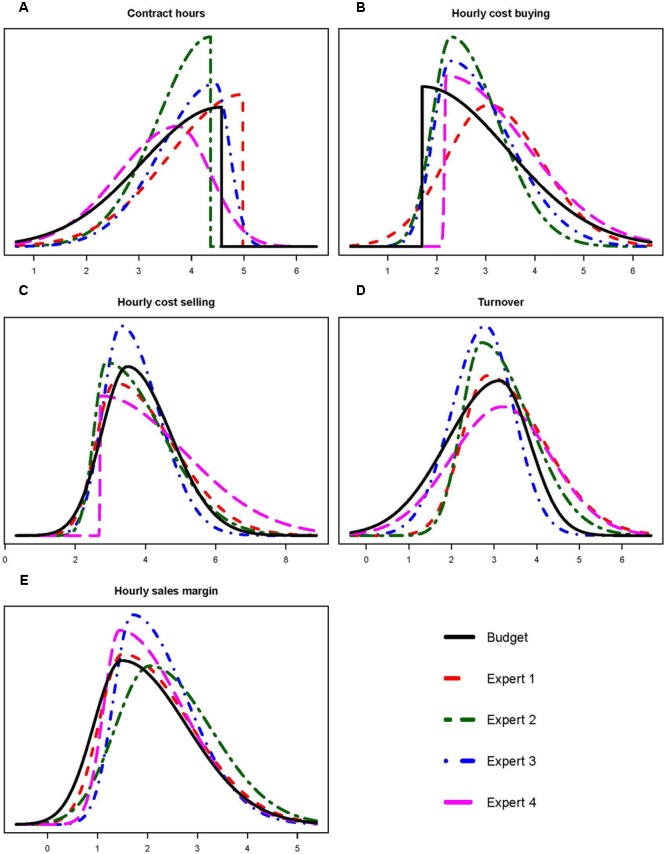
Results for elicitation with the staffing company. Experts’ predictions plotted with actual budget for contract hours **(A)**, hourly cost buying **(B)** and selling **(C)**, turnover **(D)** and hourly sales margin **(E)** concerning the first quarter of 2016.

The results can be found in **Figure 4** in which we plotted the predictions of the four experts against the actual budgets for the first quarter. To conceal the true values, which is business-sensitive information, a linear transformation has been done on all variables. It can be seen, especially for the hourly sales margins and the turnover, that experts provided very similar predictions to the budgets, for more detailed information see **Table 1**. The resemblance of the predictions to the budget indicates internal validity for the use of the steps 1 and 2 of the Five-Step Method as the elicited predictions closely match carefully constructed expectations. Based on these results we decided not to further adjust the elicitation procedure.

**Table 1:** Results for elicitation with the staffing company.

	Contract hours			Hourly cost buying			Hourly cost selling			Turnover			Hourly sales margin		
	μ	σ	γ	μ	σ	γ	μ	σ	γ	μ	σ	γ	μ	σ	γ
Expert 1	3.88	0.83	4.01^∗^10^-6^	3.21	0.99	1.10	3.99	1.14	1.73	3.39	0.98	1.48	2.18	0.94	1.68
Expert 2	3.56	0.61	4.48^∗^10^-8^	2.74	0.69	1.57	3.86	1.04	2.14	3.21	0.81	1.53	2.46	0.97	1.31
Expert 3	3.85	0.70	0.51	2.91	0.80	1.78	3.72	0.81	1.41	2.71	0.72	0.93	2.25	0.76	1.69
Expert 4	3.34	0.89	0.74	3.45	0.97	7.20	4.59	1.43	12.80	3.16	1.17	0.98	2.18	0.84	1.97
Budget	3.37	0.91	5.52^∗^10^-4^	3.09	1.05	566.00	3.87	0.99	1.29	2.71	0.99	0.76	2.06	0.96	1.51

*Experts’ predictions with actual budget for contract hours, hourly cost buying and selling, turnover and hourly sales margin concerning the first quarter of 2016.*

### Elicitation Large Financial Institution

In the third elicitation study the experts (*D* = 4) were regional directors working at a large financial institution. They are considered experts in knowledge concerning market opportunities, market dynamics and estimating the capabilities of the professionals to seize opportunities. Based on these skills we expected that they could predict the average turnover per professional in the entire country in the first quarter of 2016. In this study the experts did not predict the distribution of the data **y**, but construct a prior for the mean denoted by π_d_(𝜃). As π_d_(𝜃) ∼ SN(μ_0_,$\sigma_{0}^{2}$,γ_0_) the elicitation results in the representation of each expert’s beliefs expressed in the hyper parameters μ_0_ $\sigma_{0}^{2}$, and γ_0_. We compare the predictions of the experts against actual results, expressed as the posterior distribution of the average turnover per professional, denoted by π(𝜃|y). π(𝜃|y) ∼ SN(μ_1_,$\sigma_{1}^{2}$,γ_1_), μ_1_ denotes the posterior mean, $\sigma_{1}^{2}$ denotes the posterior variance and γ_1_ the posterior skewness. The prior for π(𝜃|y) is a N(0, 100) prior which is uninformative given the scale of the data.

#### Design

The team that participated consisted of 11 experts, 10 regional directors and one director. All were eligible to be included in the study. To comply with conditions set by the Ethics Committee, we ensured that experts whom did not wish to participate could do so without it being known that they refused. Therefore we randomly selected seven out of the 11 experts and invited them to participate. Out of the seven selected experts that we approached, three indicated that they did not want to participate in the study and four indicated that they were willing to participate. All four experts that agreed to participate, did participate and completed the elicitation. The participating experts first performed a practice elicitation for their own sales team before moving on to their estimate for the whole country, enabling them to acquaint themselves with the elicitation applications. Offering this practice elicitation could improve the quality of the elicitations (Johnson et al., 2010a). Only in the case that the director participated the practice run was be possible. The study receive ethical approval from our internal Ethics Committee of the Faculty of Social and Behavioural Sciences of Utrecht University. The letter of approval can be found in the data archive on the OSF website at https://osf.io/wvujz.

The Five-Step Method was used in this elicitation study and it consists of the following two parts: the first step is designed to support the expert in the use of reasoned and structured thoughts to obtain an estimate for the location parameter μ_0_. In the second step the estimate for μ_0_ is used and the expert is asked to provide a reasonable lower and upper bound for their estimate so the prior distribution for the mean turnover per professional can be constructed.

The “chips” placed in the first step were intended to represent individual professionals in the trial run and clusters of similar professionals in the elicitation concerning the whole country. Visual feedback was provided on the elicited distribution, accompanied by a description of the value for μ_0_ by the researcher. The expert could accept the representation of their beliefs or adjust input until the representation matched their beliefs. Results concerning country wide performance where discussed in terms of total turnover for all professionals within the team, therefore the estimate for μ_0_ was transformed using the following function

(2) $$\begin{array}{c} \theta^{*}= a\theta + b, \end{array}$$

where 𝜃 represent the parameter of interest and 𝜃 ∼ N(μ,σ^2^) so that 𝜃^∗^ ∼ N[aμ+b,(aσ)^2^].

The use of the mean as location parameter offered additional options to accommodate differences in reasoning of experts, e.g., a sales expert might feel comfortable to provide estimates for the total turnover of a store, represented by 𝜃^∗^in Eq. 2, but not be comfortable providing estimates for the mean turnover per product sold in the store, represented by 𝜃 in Eq. 2. By knowing the total amount of products that are sold in the store, entering the amount as value for a and 0 for b in Eq. 2, the prior beliefs regarding the total turnover can be transformed to prior beliefs regarding mean turnover per product and compared to predictions by other experts. The transformation procedure ensures no expert is forced to adhere to a certain scale. To illustrate this flexibility let us imagine that a store sells nine different types of products and in total sells 104 products. In steps 1 and 2 we wish to elicit and verify the location parameter for the mean turnover. Two experts feel comfortable supplying estimates for turnover per product whilst two other experts only feel comfortable supplying estimates for turnover per product type. They can both adhere to the scale they feel comfortable with as we can use a linear transformation to get them onto the same scale for steps 3 and 4. In **Table 2** we supply a numerical example to show how location parameters, elicited on a different scale, can be transformed using Eq. 2 to be on the same scale for steps three and four of the elicitation.

**Table 2:** Illustration of linear transformations using Eq. 2.

	Steps 1 and 2 product scale mean result (*n* = 104)	Steps 1 and 2 product type scale mean result (*n* = 9)	Mean turnover per product used in steps 3 and 4	Total turnover used in steps 3 and 4
Expert 1	1.8	–	1.80	187.2
Expert 2	2.1	–	2.10	218.4
Expert 3	–	23	1.99	207
Expert 4	–	24.5	2.12	220.5

*Experts 1 and 2 choose to specify turnover per product, resulting in a location parameter on the product scale. Experts 3 and 4 choose to specify turnover per product type, resulting in a location parameter on the product type scale. In steps 3 and 4 we can use either the location parameter on the total turnover scale or the mean turnover scale for experts to provide a reasonable lower and upper bound. All experts’ elicited location parameters can be transformed to both scales.*

In step 3 of the Five-Step Method, we asked the experts to provide a reasonable lower and upper bound for the total turnover of all professionals_._ Based on the input a distribution was fitted and visual feedback was provided. The researcher supported the visual feedback with a description explaining that more density on places of the axis indicate more perceived likeliness for that value. The expert could accept the representation of their beliefs or adjust the input for the reasonable lower and upper bound until the representation matched their beliefs. The elicited distribution was transformed back to represent the average turnover per professional using Eq. 2.

#### Results

During the elicitation procedures we noticed that not all experts reasoned in the same way. One expert reasoned for his own region in the expected elements, such that each “chip” represented a professional, but concerning the elicitation for the whole country the “chips” represented regional performances not clusters of professionals that are alike. This deviation did not require an adjustment of procedure just a different value for *a* in Eq. 2 to obtain the estimate for total turnover used in step 3 of the Five-Step Method. Another expert directly reasoned in total turnover when considering country wide performance and directly provided the estimate used in step 3. A third expert started, especially during the test run concerning the expert’s own team, naming the professionals aloud whilst placing the “chips,” using the expected representations for the input. The procedure proved flexible enough so that each expert could use their own careful reasoning within the same framework and end up with comparable output.

**FIGURE 5:**
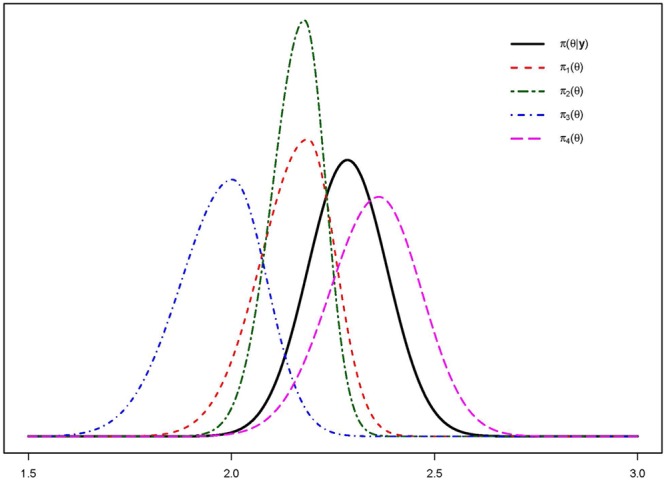
Results Five-Step Method elicitation study with large financial institution. Elicited expert distributions π_d_(𝜃) plotted with results π(𝜃|y).

All data were analyzed anonymously and were transformed to avoid revealing business-sensitive information. The elicited priors π_d_(𝜃) can be found in **Figure 5**, together with the posterior distribution π(𝜃|y). The values for the hyper parameters for π_d_(𝜃) and π(𝜃|y) can be found in **Table 3**. We can see, visually in **Figure 5** and numerically in **Table 3**, that experts one and two provide very similar predictions, however expert 2 is less uncertain about the prediction. In the same manner we can see that expert four made a prediction that closely resembles the actual realization.

**Table 3:** The values of the hyper parameters of π(𝜃|y) and π_d_(𝜃) for the study with the large financial institution.

	μ_0_	σ_0_	γ_0_	μ_1_	σ_1_	γ_1_
Preferred distribution	–	–	–	2.29	0.10	0.99
Expert 1	2.15	0.09	0.78	–	–	–
Expert 2	2.16	0.07	0.82	–	–	–
Expert 3	1.97	0.11	0.82	–	–	–
Expert 4	2.35	0.11	0.94	–	–	–

## Discussion

The Five-Step Method provides a first step for eliciting experts in a flexible manner such that no expert is forced to reason on a scale they are uncomfortable with, yet ending up with comparable priors for all experts.

In essence the Five-Step Method resembles the structure for eliciting a distribution as is proposed by Oakley (2010). Oakley states four steps, (1) the experts makes some probability judgments about the parameter of interest (2) fit a probability distribution to these judgments (3) provide feedback (4) either accept and use the distribution or repeat steps 3 and 4 based on adjusted input. The difference between what Oakley proposes and the Five-Step Method is that we repeat this cycle twice, once for the elicitation of a location parameter and once for the elicitation of the scale and shape parameters. By decomposing the elicitation task we aim to reduce bias and incorporate more feedback.

Johnson et al. (2010a) concluded that measurement properties of the elicitation methods should be evaluated. We first evaluated usability and thereafter the internal validity for the first two steps of the Five-Step Method. Companies put a lot of effort in carefully constructing their budgets. The study with the staffing company provided evidence to show that experts can produce very similar predictions to the budged using steps 1 and 2 of the Five-Step Method. This high resemblance indicates that the elicited predictions closely reflect predictions made with all available information at hand. Further indications for desirable measurement properties are found when we look at the study with the large financial institution. The data in that case are the actual realization of the average turnover per professional that the experts predicted. Using the Five-Step Method especially expert 4 provided predictions that highly overlap with the actual results, see **Figure 5**. This provides an indication for the internal validity of the method, experts are able to accurately predict future data using the Five-Step Method. To see if this result holds in general or only in our sample, and to compare the results with other elicitation methods, we recommend a larger study in which experts use multiple elicitation methods including the Five-Step Method to predict future data.

We acknowledge that asking experts for the reasonable lower and upper bound for their estimate in step 3 of the Five-Step Method could perhaps be an oversimplified procedure and other researchers might prefer to replace this step with eliciting quantiles. Goldstein and Rothschild (2014), however, found that even laypeople’s intuitions about probability distributions can become quite accurate with the help of graphical elicitation techniques. The finding by Goldstein and Rothschild in combination with our own results from the studies with the staffing company and large financial institution support us in the fact that providing graphical feedback along with the interpretation of the elicited distribution can be a key factor in the calibration of the elicitation. Obtaining confirmation from the experts that the way we represent their beliefs is justified is the crucial element in the proposed Five-Step Method. We follow the same reasoning concerning any possible anchoring bias that is introduced by first eliciting the location parameter of the prior distribution of the expert. We count on the graphical feedback along with the interpretation of the elicited distribution to ensure proper calibration of the elicited distribution. We provided some support for the internal validity of the Five-Step Method, yet to verify the external validity, and reaffirm the internal validity of the Five-Step Method a larger validation study needs to be carried out comparing the Five-Step Method with other elicitation methods.

Besides providing graphical feedback it is desirable to stay as close as possible to the reasoning experts use on a daily basis. The method should be adjusted to fit the expert’s reasoning and not the other way around if we do not want to introduce unnecessary bias. As shown in the study with the large financial institution, the Five-Step Method allows for just that. We can help experts order their thoughts, whether they reason in terms of individuals, regions or totals. All these ways of reasoning can be used by simply altering the value for a in Eq. 2 and thereafter transforming the values back to be compared on the same scale.

Using graphical feedback and flexible procedures remains a challenging task in an elicitation process. In the seminal work by O’Hagan et al. (2006) it is already recommended that user friendly software should be developed for elicitation purposes, yet each elicitation seems to require a special approach. Even so, it is a worthwhile effort to try and standardize procedures and methods as much as possible so we can work toward a situation that enables applied researchers to use elicitation procedures in their work with ease. We use the R programming language to utilize parametric fitting whilst presenting a web-based interface through the use of the shiny package. We thus use the same building blocks as MATCH. We have taken a first step to show that the Five-Step Method can aid experts in ordering and structuring their thoughts through a systematic and flexible method, tailored to each individual expert and we would welcome the adoption of the method by endeavors such as MATCH.

## Ethics Statement

This study was carried out in accordance with the recommendations of the internal Ethics Committee of the Faculty of Social and Behavioural Sciences of Utrecht University, with written informed consent from all subjects. All subjects gave written informed consent in accordance with the Declaration of Helsinki. The protocol was approved by the internal Ethics Committee of the Faculty of Social and Behavioural Sciences of Utrecht University.

## Author Contributions

DV and RvdS mainly contributed to the study design. All authors have been involved in the design of (part) of the elicitation procedure. DV programmed the elicitation software. All elicitations have been facilitated by DV and DS. DV wrote and revised the paper with feedback and input of DS, MZ-Z, and RvdS. RvdS supervised the project.

## Conflict of Interest Statement

The authors declare that the research was conducted in the absence of any commercial or financial relationships that could be construed as a potential conflict of interest.

## Footnotes

1 Using the SN distribution we represent the tacit knowledge of experts by eliciting the location parameter of the distribution, in this case the mean. The uncertainty of the expert about his/her belief on the location parameter is represented by the scale and shape parameter (i.e., variance and skewness of the normal distribution). Eliciting the mean of a normal distribution offers the advantage of easily transformable scale for elicitation procedures. An adjustable scale means that even if one expert reasons in averages and the other expert in sums they can be transformed to be comparable, i.e., let 𝜃 represent the parameter of interest and 𝜃∼N(μ,σ^2^) and if we transform 𝜃 via the following function 𝜃^∗^ = a𝜃 + b, then 𝜃^∗^∼*N*[aμ + b, (aσ)^2^].
